# Anthropometry and physical appearance can be associated with quality of life in Brazilian women with Turner syndrome

**DOI:** 10.20945/2359-3997000000535

**Published:** 2022-12-01

**Authors:** Carolina Trombeta Reis, Marina Cruvinel Macedo, André Moreno Morcillo, Gil Guerra, Sofia Helena Valente de Lemos-Marini

**Affiliations:** 1 Universidade Estadual de Campinas Campinas SP Brasil Universidade Estadual de Campinas (Unicamp), Campinas, SP, Brasil

**Keywords:** Quality of life, Turner syndrome, anthropometry, physical appearance, body, adaptation, psychological

## Abstract

**Objective:**

This study aimed to analyze if anthropometric factors and physical appearance are associated to QoL in Turner syndrome (TS).

**Materials and methods:**

Observational, analytical, and cross-sectional study. The SF-36 was applied along with an additional questionnaire regarding specific characteristics of TS.

**Results:**

There were no differences in quality of life (QoL) in TS women regarding median height and appropriate height according to parental target height, however, participants satisfied and who did not desire to change their height had better scores in the mental health and role emotional domains than those not satisfied and desired to change it. When comparing participants who were or were not bothered by physical appearance, the results showed that those not bothered by physical appearance had a better score in the vitality and social function domains. Considering patients who did or did not desire to change physical appearance, those who did not want to change their physical appearance had higher scores in the mental component and in the social function and mental health domains of the SF-36.

**Conclusion:**

This study indicated that anthropometric factors and physical appearance may possibly be associated to QoL in TS, and also emphasizes the need to develop and validate an official questionnaire regarding specific TS characteristics in order to assess in more detail how specific characteristics of TS interfere with their QoL.

## INTRODUCTION

It is commonly known that patients with chronic disorders can have Health-Related Quality of Life (HRQoL) issues, considering their physical health, social functioning, functional ability, independence, emotional functioning, and well-being (1–3).

It is crucial to analyze these patients’ HRQoL aspects not only by using general questionnaires, but by using instruments developed for specific chronic disorders, taking into consideration the most important characteristics of these conditions (4–8).

One of the most commonly used questionnaires is the Medical Outcomes Study 36 – Short-Form Health Survey (SF-36), which is a multidimensional generic health questionnaire, that has been translated into Portuguese and validated in the Brazilian population (9). This instrument has not only been used to assess the QoL associated with many chronic disorders (10–12), but has commonly been used in association with questionnaires designated for specific chronic disorders (13,14).

The SF-36 has been used to assess QoL associated with Turner syndrome (TS), which is a rare genetic condition in which women most commonly present a specific phenotype, hypogonadism, short stature, obesity, cardiac conditions and infertility, which may be associated with their QoL and emotional status (15–19).

It is essential to consider that short stature is not the only physical characteristic that can be present in patients with TS. It is known that at least 60% of them present other physical features associated with the specific TS phenotype (20), making it possible for them to have some issues regarding their physical appearance.

The SF-36 was initially developed to assess the QoL of patients with Rheumatoid Arthritis and takes into consideration some factors, such as pain, that are not usually present in TS patients. Moreover, there is no specific validated questionnaire to assess QoL in TS patients and no consensus on the literature regarding which factors can interfere the most with these patients’ well-being (21–27). Therefore, it is still crucial for medical teams to continue analyzing the QoL and psychosocial issues involved with TS more specifically.

When applying the SF-36 to 44 women with TS and 44 healthy women, a better score in the TS group was observed in relation to the control group regarding the mental component and the role physical, bodily pain, general health, social function, and emotional domains (28).

The aim of this study was to compare the results of the SF-36 with information obtained from a questionnaire regarding specific characteristics involving TS, in order to evaluate if anthropometric factors and physical appearance are associated with QoL aspects of women with TS.

## MATERIALS AND METHODS

Observational, analytical, and cross-sectional study, approved by the Research Ethics Committee of State University of Campinas (Unicamp). All the participants signed informed consent forms. The SF-36 was applied to patients with TS along with an additional questionnaire regarding specific characteristics of TS, which was developed based on the experience of professionals who work with TS patients.

The specific questionnaire developed by the authors emphasized the following aspects: level of education, professional experience, medical information and data (medication, surgeries, history and anthropometric factors), as well as satisfaction, desire to change, and being bothered or not by their height, weight and physical appearance.

The inclusion criteria included all of the TS patients from the Outpatient Clinic of the University Hospital of State University of Campinas (HC-Unicamp) aged between 18 and 30 years, who had reached their final height (growth rate less than 1 cm/year). Participants’ diagnoses were confirmed by the karyotype obtained from the medical records.

The women were monitored periodically in this service, being seen at least twice a year by the same team for a significant period (13.7 ± 6.4 years), with a minimum period of 3.7 years and a maximum of 29.9 years.

Anthropometric data on height and weight were obtained from the medical records, considering the closest date to when the research was carried out. The heights of the participants’ parents were also obtained from the medical records.

The z-score values of height, weight, and body mass index (BMI) were calculated based on data from the National Center for Health Statistics (NCHS)/Center for Disease Control and Prevention (CDC) (29) dated from 2000 and on the Pediatric Percentile Calculator for Height, Weight, BMI, and Blood Pressure program of the Quesgen Systems Incorporated (30) enterprise.

In a previous study, the authors compared the SF-36 scores of TS women to those of a control group (28). However, a control group was not used in the present study because the objective was to apply the SF-36 and a questionnaire regarding specific aspects of TS, only considering patients with this syndrome. By using both instruments, the intention of the present study was to analyze if anthropometric factors and physical appearance are associated with QoL in TS patients.

**Figure 1:**
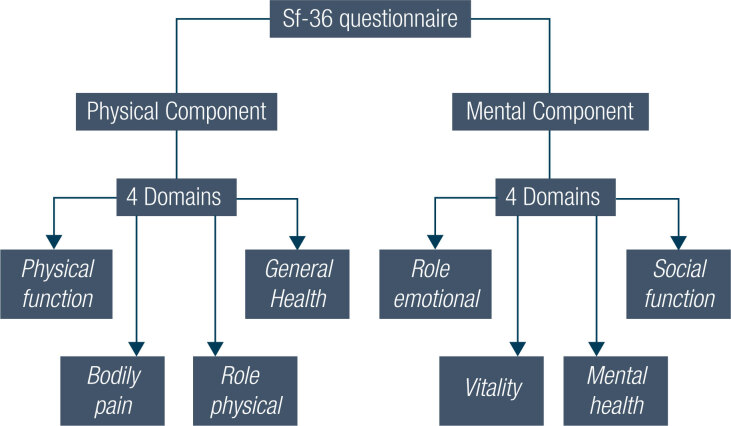
SF-36 Components and Domains (Author's personal data).

The application of the SF-36 was authorized by QualityMetric Incorporated (31), protocol QM033635. The SF-36 consists of 2 components and 8 domains that are divided as demonstrated in Figure 1 (9,31–33).

The QualityMetric Incorporated software uses algorithms that consist of numeric codes to associate responses from the questionnaire items with the scales and formulas that produce the final scores for each scale (31).

Median height, appropriate height according to parental target height and nutritional status were evaluated in relation to the SF-36, in order to determine the association between these factors and patients’ QoL.

The participants were divided in relation to the median height of the group. The individuals were separated into groups according to whether they were above or below median height, in order to evaluate the association between this factor and QoL.

Moreover, participants’ QoL was also compared in relation to having or not adequate height regarding their parental height, as well as the z-score values of the NCHS/CDC from 2000. The parental target height was calculated considering the concept proposed by Tanner and cols. in 1970 (34).


$$\begin{array}{c} \text{Parental Target Height }=\;[\text{mother's height }+\\ (\text{father's height }-\;13\text{ cm})]/2\;\pm \;8.5\text{ cm} \end{array}$$

Considering the z-score values in relation to adjustment for parental target height, the participants were divided into two groups: the first was composed of participants with adequate height in relation to the parental target height, and the second of women with height below the parental target height.

Participants were also divided considering nutritional status according to BMI, as follows: underweight (z-score ≥ −3 and < −2), normal range (z-score ≥ −2 and < 1), preobese (z-score ≥ 1 and < 2), obese class 1(z-score ≥ 2 and ≤ 3), and obese classes 2 and 3 (z-score > 3) (35). For statistical analysis, classifications of underweight and normal range were considered non-overweight, and the others were classified as overweight.

Furthermore, the SF-36 scores were compared in relation to participants being satisfied or unsatisfied with their height, weight, and physical appearance; bothered or not bothered by height, weight, and physical appearance; and with or without desire to change height, weight, and physical appearance.

As for level of satisfaction with height, weight, and physical appearance, for the statistical analysis, participants were divided in two groups: satisfied or unsatisfied with each of these items. The responses “totally satisfied”, “somewhat satisfied”, and “satisfied” were considered as satisfaction; and the responses “not completely satisfied” and “not satisfied at all” were considered as dissatisfaction. Data were statistically analyzed in order to verify whether there was a significant difference in the SF-36 scores between participants who were satisfied and unsatisfied with height, weight, and physical appearance.

Regarding being bothered by height, weight, and physical appearance, participants were asked to indicate which aspects they were unpleased with in descending order. The women were considered to be bothered with a certain item when such was marked as the aspect of greatest displeasure. For the purpose of statistical analysis, the participants were classified as those who were bothered and those who were not bothered with each item. Data were statistically analyzed in order to verify whether there was a significant difference in the SF-36 scores among participants who were bothered and those who were not bothered with height, weight, and physical appearance.

Regarding the desire to change height, weight, and physical appearance, for the purpose of statistical analysis, the participants were classified as those who desired and those who did not desire to change each of these items. The responses “I would change it a little”, “I would change it slightly”, “I would change it a lot” and “I would change it completely” were considered a desire to change, and the response “I would not change it” was classified as no desire to change. Data were statistically analyzed in order to verify whether there was a significant difference in the SF-36 scores between participants who desired and those who did not desire to change height, weight, and physical appearance.

Data processing was performed with the Statistical Package for the Social Sciences (SPSS, version 16.0, Chicago, USA) for Windows^®^. The mean, standard deviation (SD), median, minimum (min), maximum (max), and maximum amplitude of the quantitative variables were determined. The data were expressed as: mean ± SD (median; min-max). Fisher's exact test was used to assess the association between qualitative variables, and the Mann-Whitney test was employed to compare the distribution of quantitative variables. The level of significance used in all tests was 5%.

## RESULTS

There were 44 women with TS who met the inclusion criteria, and all of them consented to participate in the study. The mean age of the participants was 22.0 ± 3.6 years (21.4; 18.0-30.5), and they had a mean follow-up time in the service of 13.7 ± 6.4 years. According to schooling data, out of the 44 participants 18 had started or completed higher education and 26 had started or completed high school.

The mean height of the participants was 145.6 ± 6.4 cm (145.5; 131.0-157.5) and there was no statistically significant difference in the SF-36 scores of individuals with height above or below the median, demonstrating similar QoL in both groups.

Considering the parental target height, 10 participants had appropriate height and 34 had height below the parental target height. There was no statistically significant difference in QoL in relation to adjustment for parental target height regarding the SF-36 scores.

The mean weight of the participants was 52.0 ± 10.6 kg (32.3; 51.6-77.9). Regarding the BMI, the patients had a mean of 24.5 ± 4.4 kg/m^2^ (23.6; 16.4-35.3). A total of 32 participants were classified as non-overweight and 12 as overweight. Concerning nutritional status, there was also no statistically significant difference in QoL between groups when the SF-36 scores were analyzed.

The questionnaire developed specifically for this study demonstrated the following results among the 44 participants: 32 (satisfied with height), 25 (satisfied with weight), and 36 (satisfied with physical appearance); 33 (not bothered with height), 32 (not bothered with weight), and 39 (not bothered with physical appearance); 11 (no desire to change height), 9 (no desire to change weight), and 17 (no desire to change physical appearance). Considering the aspects of greatest displeasure, the following were stated by the participants: weight (12), height (11), physical appearance (5), infertility (11), hearing difficulty (2), and delayed puberty (1); 2 stated they were not bothered by these aspects.

Regarding the use of Human Growth Hormone (hGH), only 5 patients in our study used hGH, and their mean height was 145.4cm (140.6-152.3). All of them were satisfied with their physical appearance, and 2 were not satisfied with their height.

Regarding the use of estrogen, out of the 44 participants, 12 did not have menarche at the time of the study. Moreover, of the 8 participants who were not satisfied with their appearance, out of which 3 did not have menarche (37.5%), and considering the 36 participants who were satisfied with their physical appearance, 9 (25%) did not have menarche.

Information about each participant regarding height (cm), parental target height, BMI (kg/m^2^), menarche, uterine volume (cm^3^), hGH, satisfaction with physical appearance, and aspect of greatest displeasure has been included in Table 1.

**Table 1:** Description of height, height channel average, BMI, menarche, uterine volume, hGH, satisfaction with height, satisfaction with physical appearance and aspect of greatest displeasure

	Height (cm)	Parental Target Height (cm)	BMI (kg/m^2^)	Menarche	Uterine Volume (cm^3^)	hGH	Satisfaction with height	Satisfaction with Physical Appearance	Aspect of greatest displeasure
1	149.5	166.3	25.7	Yes	38.0	No	1	5	Physical Appearance
2	150.6	161.0	21.0	Yes	25.0	No	3	4	Infertility
3	135.0	165.9	26.1	Yes	23.0	No	4	4	Weight
4	143.0	157.3	26.8	No	10.9	No	3	4	Infertility
5	141.5	157.8	22.9	No	12.0	No	4	4	Height
6	143.5	154.5	28.6	Yes	54.7	No	3	4	Physical Appearance
7	152.0	171.5	22.4	No	6.8	No	4	4	Physical Appearance
8	144.0	150.9	16.4	Yes	37.1	No	4	4	Physical Appearance
9	148.0	160.2	23.0	Yes	45.0	No	3	3	Infertility
10	147.6	156.2	23.6	Yes	13.9	No	3	3	Hearing
11	142.2	154.8	21.0	Yes	49.0	No	3	3	Height
12	147.9	154.5	29.6	No	10.7	No	3	3	Weight
13	148.3	160.6	29.1	No	13.2	No	3	3	Hearing
14	145.9	170.4	23.3	Yes	22.0	No	4	3	Height
15	152.3	161.2	22.9	No	7.3	Yes	3	3	Physical Appearance
16	143.2	157.5	18.6	Yes	29.2	No	4	3	Height
17	142.4	154.4	25.7	Yes	20.0	Yes	4	3	Height
18	154.0	148.0	25.8	Yes	26.0	No	3	3	Infertility
19	148.0	164.5	24.7	Yes	17.8	No	3	3	Infertility
20	143.2	162.7	23.5	No	12.0	No	3	3	Height
21	140.2	159.8	23.2	Yes	33.1	No	4	3	Height
22	142.0	157.0	20.0	Yes	47.9	No	3	3	Infertility
23	151.5	159.7	24.7	Yes	24.0	No	1	3	Weight
24	139.0	148.4	16.7	Yes	21.8	No	3	3	Infertility
25	150.0	157.3	23.6	Yes	27.8	No	3	3	Weight
26	140.6	153.5	26.3	Yes	18.0	Yes	3	3	Weight
27	139.5	159.8	31.2	No	0.9	No	5	3	Infertility
28	148.5	164.4	35.3	Yes	18.0	Yes	3	3	None
29	155.0	154.5	21.0	Yes	41.0	No	3	2	Height
30	156.0	164.6	24.2	No	10.4	No	5	2	Height
31	153.2	161.2	30.5	Yes	15.0	No	2	2	Hearing
32	137.6	153.4	25.4	Yes	35.6	No	3	2	Weight
33	134.8	156.4	23.0	Yes	38.0	No	2	2	Height
34	146.0	160.2	19.8	Yes	54.4	No	3	2	Infertility
35	143.0	161.0	20.3	Yes	32.1	Yes	4	2	Height
36	134.0	155.3	24.5	No	5.0	No	3	2	Weight
37	149.7	164.2	31.5	Yes	21.4	No	1	1	Weight
38	144.5	160.1	20.5	No	10.0	No	3	1	Delayed Puberty
39	137.8	150.3	33.2	Yes	21.0	No	3	1	Weight
40	131.0	154.0	19.6	Yes	43.0	No	5	1	None
41	157.5	167.8	29.2	Yes	12.0	No	1	1	Weight
42	148.7	155.5	23.6	Yes	30.4	No	2	1	Weight
43	157.3	154.3	17.7	Yes	86.0	No	1	1	Weight
44	145.0	168.5	30.9	No	8.3	No	3	1	Infertility

1 Totally satisfied.

2 Somewhat satisfied.

3 Satisfied.

4 Not completely satisfied.

5 Not satisfied at all.

As for satisfaction with height, the 32 satisfied participants had a mean height of 146.6 cm (134.0-157.5), and the 12 unsatisfied participants had a mean height of 142.8 cm (131.0-156.0). There were no significant differences in QoL between groups, considering the SF-36's physical and mental components. In the SF-36 scores, there was a difference in the mental health domain between groups, and participants satisfied with their height showed better scores (p = 0.013) (Table 2).

**Table 2:** SF-36 scores regarding satisfaction with height

	Satisfied with height (n = 32)	Unsatisfied with height (n = 12)	p*
Physical function	95.0 (55.0-100.0)	95.0 (55.0-100.0)	0.978
Role physical	100.0 (50.0-100.0)	100.0 (50.0-100.0)	0.444
Bodily pain	84.0 (51.0-100.0)	84.0 (51.0-100.0)	0.836
General health	84.5 (17.0-100.0)	77.0 (67.0-100.0)	0.884
Vitality	75.0 (50.0-100.0)	62.5 (40.0-100.0)	0.130
Social function	100.0 (62.5-100.0)	87.5 (12.5-100.0)	0.107
Role emotional	100.0 (33.3-100.0)	100.0 (0.0-100.0)	0.793
Mental health	82.0 (56.0-100.0)	70.0 (32.0-100.0)	**0.013**

* Mann-Whitney Test. Data were expressed as median, minimum, and maximum.

Concerning being bothered by height, there was no significant difference in QoL between groups, considering the SF-36 scores. As for desire to change height, there were no significant differences in QoL regarding the SF-36's physical and mental components. Considering the SF-36 scores, there was a difference in the role emotional domain between groups, and participants who did not desire to change their height showed better scores (p = 0.032) (Table 3).

**Table 3:** SF-36 scores regarding desire to change height

	Desire to change height (n = 33)	Do not desire to change height (n = 11)	p*
Physical function	95.0 (55.0-100.0)	95.0 (80.0-100.0)	0.577
Role physical	100.0 (50.0-100.0)	100.0 (50.0-100.0)	0.431
Bodily pain	84.0 (51.0-100.0)	84.0 (72.0-100.0)	0.571
General health	82.0 (17.0-100.0)	87.0 (62.0-100.0)	1.000
Vitality	70.0 (40.0-100.0)	75.0 (50.0-100.0)	0.185
Social function	100.0 (12.5-100.0)	100.0 (62.5-100.0)	0.951
Role emotional	100.0 (0.0-100.0)	100.0 (66.7-100.0)	**0.032**
Mental health	80.0 (32.0-100.0)	84.0 (72.0-100.0)	0.057

* Mann-Whitney Test. Data were expressed as median, minimum, and maximum.

There was no significant difference in QoL between groups considering SF-36 scores when analyzing satisfaction with weight, being bothered by weight, and desire to change weight.

As for satisfaction with physical appearance, there was also no significant difference in QoL between groups, considering the SF-36 scores. Concerning being bothered by physical appearance, there were no significant differences in QoL considering the SF-36 physical and mental components. However, there were differences in the vitality and social function domains between both groups, in which participants who were not bothered with physical appearance had better scores (p = 0.037 and p = 0.025, respectively) (Table 4).

**Table 4:** SF-36 scores regarding being bothered by physical appearance

	Bothered by physical appearance (n = 5)	Not bothered by physical appearance (n = 39)	p*
Physical function	95.0 (75.0-100.0)	95.0 (55.0-100.0)	0.849
Role physical	100.0 (50.0-100.0)	100.0 (50.0-100.0)	0.481
Bodily pain	84.0 (51.0-100.0)	84.0 (51.0-100.0)	0.892
General health	72.0 (67.0-82.0)	87.0 (17.0-100.0)	0.131
Vitality	60.0 (40.0-80.0)	75.0 (40.0-100.0)	**0.037**
Social function	87.1 (12.5-100.0)	100.0 (62.5-100.0)	**0.025**
Role Emotional	100.0 (0.0-100.0)	100.0 (33.3-100.0)	0.197
Mental health	72.0 (44.0-80.0)	80.0 (32.0-100.0)	0.117

* Mann-Whitney Test. Data were expressed as median, minimum, and maximum.

As for desire to change physical appearance, there was a significant difference in QoL between groups in the SF-36 mental component (p = 0.011) and there were also differences in the social function and mental health domains; in both of them, participants who did not desire to change their physical appearance had higher scores (p = 0.006 and p = 0.006, respectively) (Table 5).

**Table 5:** SF-36 scores regarding desire to change physical appearance

	Desire to change physical appearance (n = 27)	Do not desire to change physical appearance (n = 17)	p*
Physical function	95.0 (55.0-100.0)	95.0 (80.0-100.0)	0.072
Role physical	100.0 (50.0-100.0)	100.0 (75.0-100.0)	0.336
Bodily pain	84.0 (51.0-100.0)	84.0 (51.0-100.0)	0.457
General health	82.0 (67.0-100.0)	77.0 (17.0-100.0)	0.771
Vitality	70.0 (40.0-85.0)	75.0 (60.0-100.0)	0.109
Social function	87.5 (12.5-100.0)	100.0 (62.5-100.0)	**0.006**
Role emotional	100.0 (0.0-100.0)	100.0 (66.7-100.0)	0.236
Mental health	76.0 (32.0-92.0)	84.0 (60.0-100.0)	**0.006**

* Mann-Whitney Test. Data were expressed as median, minimum, and maximum.

## DISCUSSION

There was no difference in QoL among women with TS with height above or below the median of the group, with height either adjusted or not for the parental target, and between participants classified as overweight or non-overweight. However, when analyzing the responses to the questionnaire developed specifically regarding TS characteristics, it was observed that participants unsatisfied with their height had worse scores in the mental health domain, and those who desired to change their height had worse scores in the role emotional domain. Moreover, participants bothered by their physical appearance had worse scores in the vitality and social function domains, and those who desired to change their physical appearance had lower scores in the mental component as well as the social function and mental health domains of the SF-36.

Our findings regarding height are similar to those obtained by Taback and Van Vliet (2011) (24). The authors used the SF-36 questionnaire and found no difference in QoL when comparing two groups of women with TS, one of which had been treated with hGH and one that had not. The first group consisted of 21 participants with mean height of 148.9 cm, and the second of 12 patients with mean height of 143.7 cm. Likewise, authors of a study conducted in France with 568 TS patients divided into three groups according to height (>152.1 cm, between 146.5 and 152.1 cm, and < 146.5 cm) found no difference in QoL assessed by SF-36 between taller and shorter participants (21).

Authors of other studies identified differences in QoL of adult women with TS and verified better scores in taller women among the evaluated groups: in the physical function, role physical, vitality, and bodily pain domains (23), as well as the physical function domain (25), both using the SF-36 questionnaire.

However, although the participants of our study with height below median or below the growth channel do not have a QoL worse than participants with height above such values, evaluating the questionnaire responses enables one to observe that participants satisfied with height had better scores in the mental health domain than unsatisfied participants. Such a difference was not verified in another study that found better scores only in the physical function domain of the SF-36 and in the scale used for assessing the gross motor function of the instrument TNO/AZL Adult Quality of Life (TAAQOL) in a group of women with TS (22).

In the present study, participants who did not desire to change their height also had better scores in the role emotional domain, which may emphasize the association between height and QoL,

It is also important to consider that other aspects might be involved in the perception of satisfaction, desire to change, or being bothered by their height, such as parental target height, hGH, estrogen, and physical stigmas. Taking this into consideration, the use of hGH and estrogen were analyzed in some aspects, and the mean height of the 5 patients who used hGH was only 0.2 cm higher than the mean height of the whole group of participants. As for the use of estrogen, out of the 12 participants who did not have menarche at the time of the study, six had a uterine volume higher than 10, demonstrating the effects of estrogen. It is also important to consider that out of the 44 participants 31 used estrogen replacement therapy, 13 had spontaneous puberty, and 3 were mothers.

The present study also analyzed the relation between physical appearance and QoL. Participants not bothered with their physical appearance had better scores in the vitality and social function domains. Those who did not desire to change their physical appearance had higher scores in the mental component and in the social function and mental health domains of the SF-36.

Regarding physical appearance and QoL in TS patients, a study reported that participants who had feelings of insecurity due to their physical appearance had worse results in the social function domain of the SF-36 and TAAQOL, considering a probable association between physical appearance and QoL in TS, as observed in our study (22). Authors of another study, who specifically assessed QoL related to the prevalence of skeletal anomalies, found worse scores in the vitality domain in individuals with this type of condition (25).

It must be considered that since the participants of our study have been seen about twice a year by the same healthcare team for a long period and have frequent contact with other TS women, such factors may have favored the best adjustment regarding their clinical characteristics.

The main results of the present study led us to discuss that height and physical appearance may be associated with QoL in women with TS. Although height was not determined to be a significant factor regarding QoL in TS, this study made it possible to notice that this aspect, as well as physical appearance, must be better analyzed in future studies.

It is important to mention that the SF-36 was not developed specifically to analyze QoL in TS; this instrument was chosen for this study because it is currently the most used instrument for assessing QoL in this population (18). It is noteworthy that due to factors such as variability in chromosomal constitution, clinical profile, and TS phenotype, the SF-36 may not be the most suitable instrument to assess QoL in TS patients, as it does not address specific characteristics of TS such as short stature and dysmorphisms.

Regarding the study's limitations, it was not possible to use a greater number of participants; nevertheless, all women who met the inclusion criteria and were seen at the hospital where the study was conducted were evaluated.

Data from literature on QoL in TS patients are contradictory, and it is not possible to determine whether women with TS have better or worse QoL and which factors interfere with this aspect. Due to the great difficulty that occurred in analyzing these issues, it is essential to perform more studies on such topic and develop a questionnaire to better evaluate QoL in TS, considering their specific characteristics, and thoroughly analyze which factors related to TS interfere in these women's QoL.

In conclusion, participants satisfied with their height showed significantly better scores in the mental health domain of the SF-36 than those who were unsatisfied with their height. Those who did not desire to change their height had significantly better scores in the role emotional domain than those who desired to change it. Physical appearance is also probably a factor associated with the participants’ QoL, considering the higher scores in the vitality and social function domains of participants who were not bothered with their physical appearance. Participants who did not desire to change their physical appearance had higher scores in the mental component and in the social function and mental health domains of the SF-36 than those who desired to change it.

Considering that several chronic diseases have specific QoL instruments and several different aspects that might influence the QoL of TS patients, this study has demonstrated the importance of developing and validating an official questionnaire in order to assess in more detail how specific characteristics of TS interfere with these women's QoL.
